# SARS-CoV-2 Nsp1 Is a Major Suppressor of HLA Class I and Class II Expression

**DOI:** 10.3390/v17081083

**Published:** 2025-08-05

**Authors:** Ivo Schirmeister, Nicolas Eckert, Sebastian Weigang, Jonas Fuchs, Lisa Kern, Georg Kochs, Anne Halenius

**Affiliations:** 1 Institute of Virology, Medical Center University of Freiburg, 79104 Freiburg, Germany; ivo.schirmeister@uniklinik-freiburg.de (I.S.);; 2 Faculty of Medicine, University of Freiburg, 79104 Freiburg, Germany; 3 Faculty of Biology, University of Freiburg, 79104 Freiburg, Germany

**Keywords:** MHC, ORF8, Nsp1

## Abstract

Human leukocyte antigen class I (HLA-I) molecules present intracellular peptides on the cell surface to enable CD8+ T cells to effectively control viral infections. Many viruses disrupt this antigen presentation pathway to evade immune detection. In this study, we demonstrate that SARS-CoV-2 Nsp1 impairs both the constitutive and interferon-γ (IFN-γ)-induced upregulation of HLA-I. Moreover, Nsp1 also blocks IFN-γ-induced expression of HLA-II. We found that, contrary to previously published work, the early SARS-CoV-2 B 1.1.7 Alpha variant lacking the accessory protein ORF8 retained full capacity to downregulate HLA-I, comparable to an ORF8-expressing wild-type isolate. While ectopic overexpression of ORF8 could reduce HLA-I surface levels, this effect was only observed at high expression levels. In contrast, moderate expression of the viral protein Nsp1 was sufficient to potently suppress both basal and IFN-γ-induced HLA-I, as well as HLA-II expression. To probe the underlying mechanism, we analyzed HLA-I-associated genes in previously published RNA-sequencing datasets and confirmed that Nsp1 reduces expression of components required for HLA-I biosynthesis and antigen processing. These findings identify Nsp1 as a key factor that impairs antigen presentation pathways, potentially contributing to the ability of SARS-CoV-2 to modulate immune recognition.

## 1. Introduction

Severe acute respiratory syndrome coronavirus 2 (SARS-CoV-2) is the causative agent of the coronavirus disease 2019 (COVID-19) and is responsible for the pandemic, which has caused more than 760 million cases with almost 7 million fatalities. SARS-CoV-2 is a positive-sense, single-stranded RNA virus and belongs to the genus *Betacoronavirus* [1], which also includes the two highly pathogenic viruses to humans, SARS-CoV [2] and Middle East respiratory syndrome coronavirus (MERS-CoV) [3].

Although adaptive immune responses are elicited following SARS-CoV-2 infection, they often fail to provide sterilizing immunity against emerging variants. Neutralizing antibodies targeting the spike protein lose efficacy as the virus evolves [4], whereas CD8+ and CD4+ T-cell responses remain relatively conserved and play a crucial role in limiting viral replication and reducing disease severity [5,6,7,8,9]. CD8+ T cells recognize viral peptides presented on MHC-I molecules, while CD4+ T cells respond to peptides presented on MHC-II and orchestrate broader immune responses. These pathways are therefore important targets for viral immune evasion.

In addition to the structural proteins spike (S), envelope (E), membrane (M), and nucleocapsid (N), the SARS-CoV-2 genome encodes for several accessory proteins. Many of these have been shown to possess immunoevasive functions [10,11,12] and to interfere with MHC-I antigen presentation. It was reported that ORF8 degrades MHC-I in human and mouse cells in a lysosome-dependent manner [13]. ORF6 targets transcription and function of NLRC5, a key transcriptional regulator of MHC-I expression [10], and ORF7a and ORF3a impair surface expression of MHC-I through effects on β2-microglobulin (β_2_m) binding and protein trafficking, respectively [14].

Moreover, SARS-CoV-2 infection leads to a global suppression of host gene expression [15], largely driven by the nonstructural protein Nsp1, a critical virulence factor [16]. Nsp1 inhibits host translation by stalling initiation and promoting degradation of host transcripts, thereby blocking interferon (IFN)-induced gene expression [17,18,19,20,21]. Viral transcripts evade this suppression [22,23,24], allowing continued viral protein synthesis.

While several SARS-CoV-2 proteins have been shown to inhibit MHC-I antigen presentation, direct analyses of this process in infected cells remain limited. To better understand how SARS-CoV-2 modulates antigen presentation during infection, we examined MHC-I surface expression in infected cells and observed a pronounced downregulation. Unexpectedly, this suppression occurred independently of ORF8 and extended to both basal and IFNγ-induced expression of MHC-I. Further investigation revealed that ORF8 could reduce HLA-I surface density only under conditions of transient, high-level overexpression. In contrast, Nsp1 potently suppressed both basal MHC-I and IFNγ-induced expression of MHC-I and MHC-II, consistent with its established role in global translational shutoff [17,19]. Collectively, our findings position Nsp1 as a major SARS-CoV-2 inhibitor of MHC-I and MHC-II antigen presentation, suggesting a key mechanism by which the virus evades T-cell mediated immunity.

## 2. Material and Methods

### 2.1. Molecular Cloning

SARS-CoV-2 encoded genes were amplified by PCR from wild-type SARS-CoV-2 cDNA (Table 1). PCR products were digested with NheI and BamHI restriction enzymes (New England Biolabs). The purified fragments were subsequently cloned into the NheI and BamHI restriction sites of the following expression vectors: pIRES-EGFP (MIEP promoter) and pucIP and pucGFP (SFFV U3 promoter), described previously [25]. Control constructs encoding HA-US11 and RL8 from HCMV were described previously [25].

### 2.2. Cell Culture and Protein Overexpression

Calu-3 (ATCC HTB-55, kindly provided by Markus Hoffmann, Göttingen, Germany), HEK293T (ATCC: CRL-3216), HeLa (ATCC: CCL-2), and MRC-5 (ATCC: CCL-171) cells were cultured in DMEM supplemented with 10% (*v*/*v*) fetal calf serum and 50 U/mL penicillin–streptomycin at 37 °C in 5% CO_2_. Expression plasmids were transiently transfected into HEK293T and HeLa cells using polyethyleneimine (PEI MAX 40K; Polysciences Inc., Warrington, PA, USA) according to the manufacturer’s instructions.

Lentiviruses were produced as previously described [26]. Transduced HEK293T and HeLa cells were selected by puromycin (5 μg/mL) for 4–5 days. MRC-5 fibroblasts were transduced at 70% confluency.

### 2.3. SARS-CoV-2 Viruses and Infection

Infection experiments with SARS-CoV-2 were conducted under Biosafety Level 3 (BSL-3) conditions at the Institute of Virology, Freiburg, with approval from the Regierungspraesidium Tuebingen (Permit Nos. 25-27/8973.10-18 and UNI.FRK.05.16-29). We used two SARS-CoV-2 isolates: the prototypic strain Muc-IMB-1, lineage B.1, kindly provided by Roman Woelfel from the Bundeswehr Institute of Microbiology [27], and the Alpha variant B.1.1.7 (hCoV-19/Germany/NW-RKI-I-0026/2020; GISAID ID: EPI_ISL_751799), provided by Donata Hoffmann and Martin Beer (Friedrich-Loeffler-Institute, Riems, Germany). Sequencing of the B.1.1.7 isolate was described previously [28].

Approximately 0.5 × 10^6^ Calu-3 (HTB-55) cells were infected with an MOI of 0.5 in 6-well plates for 2 h at 37 °C with 1 mL OptiMEM containing the virus with or without IFNγ (ca 700 U/mL, Cat#570208, Biolegend, San Diego, CA, USA). Then, cells were washed with PBS, and 2 mL DMEM containing 2% FCS, with or without IFNγ, was added again. The IFNy concentration was chosen according to a previously established protocol [29]. At 30 h post-infection, the cells were harvested using trypsin.

### 2.4. Antibodies

The following antibodies were used for flow cytometry: W6/32-APC (Biolegend, San Diego, CA, USA, binds to β_2_m-assembled HLA-I [30]), HLA-Bw6-APC (130-099-857, Miltenyi, Bergisch Gladbach, Germany), anti-CD71-APC (clone AC102, Miltenyi, Bergisch Gladbach, Germany), anti-HLA-II-APC (clone REA332, Miltenyi, Bergisch Gladbach, Germany), anti-N (anti-SARS-CoV-2 nucleocapsid, 200-401-A50, Rockland Immunochemicals, Limerick, PA, USA), anti-HA-APC (clone GG8.1F3.1.1, Miltenyi, Bergisch Gladbach, Germany), anti-HLA-A*02-APC (clone BB7.2, Biolegend, San Diego, CA, USA), anti-HLA-C-APC (clone DT.9, Biolegend, San Diego, CA, USA), goat anti-mouse-APC (550826, BD Biosciences, Franklin Lakes, NJ, USA), and anti-IgG-Alexa488 (A11008, Invitrogen, Carlsbad, CA, USA).

The following antibodies were used for Western blotting and immunoprecipitation: anti-ORF8 (SARS-CoV-2; DU 68491; MRC-PPU, University of Dundee, Dundee, UK), HC10 (binds to the heavy chain of HLA-I [31]), anti-β-actin (A2228, Sigma-Aldrich, St. Louis, MO, USA), anti-HA (H3663, Sigma-Aldrich, St. Louis, MO, USA), anti-N (SARS-CoV-2, 200-401 A50, Rockland Immunochemicals, Limerick, PA, USA), anti-ORF3a (SARS-CoV-2, 34340S, Cell Signaling Technology, Danvers, MA, USA), anti-Nsp1 (SARS-CoV-2, 57896S, Cell Signaling Technology, Danvers, MA, USA), anti-GFP (sc-9996, Santa Cruz Biotechnology, Dallas, TX, USA), goat-anti-mouse-POD (115-035-146, Dianova GmbH, Hamburg, Germany), and goat-anti-rabbit POD (100-335-8441, Sigma-Aldrich, St. Louis, MO, USA).

### 2.5. Flow Cytometry

All cell lines were trypsinized and washed and stained in PBS containing 3% (*v*/*v*) FCS. Cells were analyzed using a FACS Canto II or FACS Fortessa flow cytometer (Becton, Dickinson and Company/BD Biosciences, Franklin Lakes, NJ, USA). In SARS-CoV-2 infection experiments, cells were fixed with 4% paraformaldehyde for 15 min on ice. For intracellular N-staining, cells were permeabilized in PBS containing 10% (*v*/*v*) FCS and 0.1% saponin for 5 min at room temperature. All subsequent staining steps were performed on ice using the same buffer.

Flow cytometry data were analyzed using FlowJo software (v10.1; Tree Star Inc., Ashland, OR, USA). Statistical analysis was performed in Prism 8 (GraphPad Software, LLC, Boston, MA, USA) using one-way ANOVA. *p*-values were considered significant as follows: *p* < 0.05 (*), *p* < 0.005 (**), *p* < 0.0005 (****), and *p* < 0.0001 (****).

### 2.6. Western Blotting

For Western blot analysis, cells were trypsinized at 24 h post-transfection, washed twice with ice-cold PBS, and resuspended in 1× lysis buffer (Luciferase Reporter Gene Assay, 11814036001, Roche Applied Science, Penzberg, Germany) and incubated for 60 min at –20 °C. After thawing on ice, cellular debris was removed by centrifugation, and the supernatant was mixed with a denaturing buffer (final concentration: 0.5% SDS and 40 mM DTT) prior to heat treatment (95 °C for 5 min). Proteins were separated by SDS-PAGE and transferred to a nitrocellulose membrane (Amersham). After incubation with the primary antibody, a secondary peroxidase-coupled antibody was applied. Chemiluminescence was detected using a LI-COR Odyssey blot scanning system, and images were processed with Image Studio software (version 5.5).

For analysis of SARS-CoV-2-infected cells, samples were directly lysed in the culture vessel using Tissue Protein Extraction Reagent (T-PER; Thermo Fisher Scientific, Waltham, MA, USA) supplemented with SDS and β-mercaptoethanol. Lysates were heat-treated twice at 95 °C for 5 min and analyzed as described above.

### 2.7. Analysis of Published RNA-Seq Datasets

The scRNA-seq data of human bronchial epithelial cells (HBECs) were downloaded under accession number GSE166766 [32]. Quality control and downstream analysis were performed using Seurat v.4.3.0 [33]. As described by Ravindra et al. [32], cells expressing >10% mitochondrial genes were excluded before downstream analysis, together with cells displaying a total number of transcripts <1500 or >7000. Cell type annotations and infection threshold were adopted from Ravindra et al. Count data of the filtered cells were normalized and scaled. To remove batch effects between mock and infected samples, as observed by Ravindra et al., a batch correction with the Harmony package (v.0.1.1) was performed [32,34]. Default parameters were used to run Seurat. Dimensionality reduction for visualization was performed using RunUMAP function, where reduction parameter was set to “harmony.” For further analyses, all cells besides ciliated, BC/club, and club cells were excluded, as they barely displayed successful infection. DotPlot function was used to visualize scaled expression of genes associated with MHC-regulation.

We analyzed mRNA reads from Fisher et al. [17] derived from the SARS-CoV-2 infection of Calu3 cells. Noam Stern-Ginossar and Tal Fisher (Weizmann Institute of Science, Israel) generously shared already processed data according to the original publication, including reads per kilobase of transcript, per million mapped reads (RPKM) normalization. For our analysis, we only included data from virus-infected cells collected at 7 h post-infection.

## 3. Results

### 3.1. SARS-CoV-2 Efficiently Controls Surface HLA-I Levels on Infected Cells

**Figure 1:**
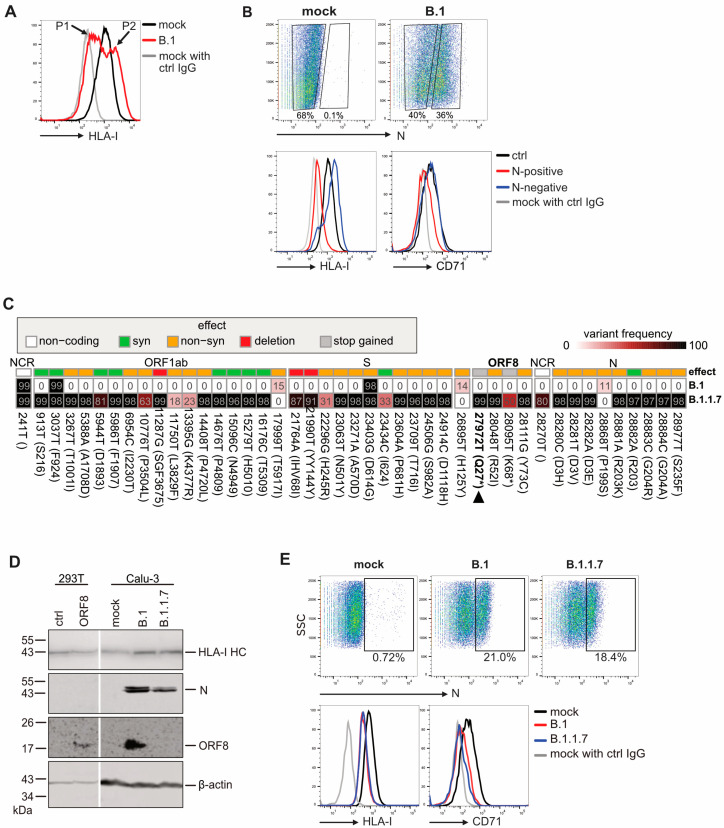
SARS-CoV-2 efficiently controls surface HLA-I levels on infected cells. (**A**) Calu-3 cells were mock-treated or infected with SARS-CoV-2 (B.1) with an MOI of 0.5. At 30 h p.i., cell surface expression of HLA-I was determined by flow cytometry analysis. P2 represents uninfected bystander cells, while P1 represents infected cells. (**B**) A flow cytometry analysis was performed as in A, including intracellular staining of N. (**C**) Genome comparison of B.1 and B1.1.1.7. Nucleotide alterations are depicted according to the indicated colour scheme. An asterisk denotes a change to a stop codon (**D**) Calu-3 cells were infected with the B.1 and B1.1.1.7 variants at an MOI of 0.5. At 30 h p.i., protein expression was determined by Western blot analysis. HEK293T cells transiently transfected with a plasmid encoding ORF8 were included as a control. (**E**) Calu-3 cells were infected, as in D, and a flow cytometry analysis was performed, as described in B, with indicated antibodies.

To evaluate the impact of SARS-CoV-2 on the MHC-I antigen presentation pathway, we analyzed human leukocyte antigen class I (HLA-I) cell surface expression on SARS-CoV-2-infected cells. Human lung epithelial (Calu-3) cells were infected with an early isolate of SARS-CoV-2 (B.1) at an MOI of 0.5, and at 30 h post-infection, HLA-I was analyzed by flow cytometry (Figure 1A). CD71 (transferrin receptor) was included as a control protein, representing a cell surface glycoprotein. Notably, we observed two HLA-I peaks in the histogram of the Calu-3 cells (P1 and P2). Co-staining with an anti-N antibody identified N-positive B.1-infected cells with reduced HLA-I surface expression (corresponding to P1), while uninfected (N-negative) cells showed increased HLA-I (P2) expression (Figure 1B).

It was previously reported that the SARS-CoV-2 accessory protein ORF8 induces degradation of HLA-I molecules by redirecting them to the lysosomal compartment [13]. Genetic analysis of an early SARS-CoV-2 variant of concern, the Alpha variant B 1.1.7 [28], identified a premature stop codon at position 27 of ORF8 (Q27*, Figure 1C), which we confirmed by Western blot analysis (Figure 1D). This natural ORF8 truncation provided an opportunity to assess the role of ORF8 in HLA-I regulation in SARS-CoV-2-infected cells. In Western blot analysis, total levels of HLA-I heavy chain (HC) appeared elevated in SARS-CoV-2-infected cells, irrespective of ORF8 expression. We considered it likely that HLA-I upregulation in non-infected bystander cells contributed to the overall increased HC signal, thereby masking a possible downregulation induced by SARS-CoV-2. Therefore, next, we monitored HLA-I surface expression in the N-positive cell population by flow cytometry. Surprisingly, regardless of the virus isolate, HLA-I levels were reduced by approximately 40% compared to mock-treated cells (Figure 1E). These findings indicated that SARS-CoV-2 infection strongly downregulated HLA-I at the plasma membrane independently of ORF8. CD71 was reduced about 30% (Figure 1E).

### 3.2. SARS-CoV-2 Blocks IFNγ-Induced Surface Expression of HLA-I and HLA-II

**Figure 2:**
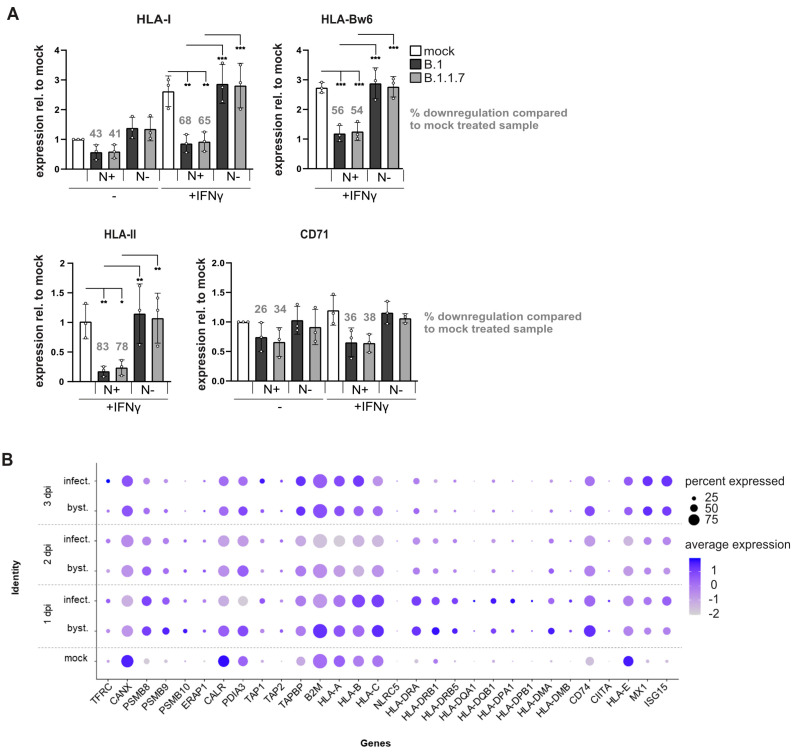
SARS-CoV-2 blocks IFNγ-induced surface expression of HLA-I and HLA-II. (**A**) Calu-3 cells were infected with B.1 or B1.1.1.7 with an MOI of 0.5. Simultaneously, IFNγ was applied to the cells. At 30 h p.i., cells were analyzed by flow cytometry with antibodies as indicated. Cell populations were gated according to an intracellular N-staining. Fold change of surface expression on N-positive and N-negative cells was calculated as the ratio of mean fluorescence intensity (MFI), as compared to mock-treated cells. Representative histograms are shown in Supplementary Material Figure S1A. Dots represent individual values, and bars represent mean values ± SD from three independent experiments. Statistical significance compared to mock-treated control cells was assessed using a one-way ANOVA (N = 3). Only comparisons with *p* < 0.05 are marked in the figure at *p* < 0.05 (*), *p* < 0.01 (**), *p* < 0.001 (***). (**B**) Regulation of HLA-associated genes in the scRNA-seq dataset of human bronchial epithelial cells [32]. Dot plot of selected genes is displayed among mock-infected (mock), bystander (byst.), and infected cells (infect.) 1, 2, and 3 days post-infection (dpi). Scaled expression across ciliated, BC/club, and club cells is depicted.

Since SARS-CoV-2 increases IFNγ signaling pathways in infected individuals [35], thereby inducing an antiviral state and increased HLA-I expression, we investigated SARS-CoV-2 regulation of HLA-I in the presence and absence of IFNγ. At 30 h post-infection (MOI 0.5), the IFNγ-induced HLA-I expression was effectively suppressed by both B.1 and B.1.1.7 variants, reducing surface expression by 56% and 54%, respectively, compared to IFNγ-stimulated mock-treated cells (Figure 2A). Hence, on IFNγ-treated N-positive cells, HLA-I cell surface levels remained similar to non-stimulated mock-treated cells. These findings indicate that in SARS-CoV-2-infected cells, IFNγ-induced biosynthesis of HLA-I is effectively blocked, likely resulting in a substantial impairment of HLA-I presentation of newly synthesized viral peptides.

Since IFNγ induces HLA-II expression in Calu-3 cells, we examined whether this response is affected by SARS-CoV-2 infection. HLA-II surface levels were reduced approximately 80% compared to IFNγ-stimulated, mock-treated cells (Figure 2A).

To investigate whether SARS-CoV-2 can control the transcription of genes associated with HLA expression, we analyzed a published single-cell RNA-sequencing dataset from SARS-CoV-2-infected human lung organoids [32], which included bronchiolar epithelial types such as club cells, basal cells, and a BC/Club cluster previously shown to correspond to bipotent basal/club progenitors at bronchial junctions. We focused our analysis on ciliated epithelial cells, BC/club cells, and club cells (Supplementary Material Figure S1B), the primary targets of infection in that system, comparing infected cells, non-infected bystander cells, and mock-treated controls. Most IFN-inducible genes (e.g., HLA-I and HLA-II encoding genes, *TAP1/2*, and *PSMB8/9*) showed increased transcription in both infected and bystander cells compared to mock-treated controls (Figure 2B), consistent with activation of antiviral signaling, as further supported by upregulation of *MX1* and *ISG15*. However, gene induction was generally lower in infected cells than in bystander cells, suggesting a dampened response in these cells. This effect was pronounced for β2-microglobulin (*B2M*), but reductions in the mRNA levels of the lectin-like chaperone calreticulin (*CALR*) and the thiol oxidoreductase ERp57 (*PDIA3*), both of which are non-interferon-responsive, were also observed following SARS-CoV-2 exposure. These proteins are important for the proper folding and assembly of HLA-I in the ER. These results indicate that SARS-CoV-2 reduces expression of key ER-resident chaperones, potentially disrupting also HLA-I antigen presentation.

### 3.3. Cells Stably Expressing ORF8 Do Not Regulate HLA-I

An ORF8-mediated effect on HLA-I was not observed in SARS-CoV-2-infected cells. However, since SARS-CoV-2 and SARS-CoV-2-ΔORF8 not only exhibit differences in the ORF8 gene (Figure 1C), potential effects of ORF8 may be masked by other changes in the genome. To assess the function of ORF8, we stably expressed ORF8 with and without a C-terminal HA-epitope tag in HEK293T and HeLa cells. In both cases, ORF8 expression did not alter the total HLA-I heavy chain (HC) level (Supplementary Material Figure S2A). As a control, cells expressing HA-US11, an HA-tagged human cytomegalovirus protein that downregulates HLA-I via the ER-associated degradation pathway [36], exhibited strongly reduced HLA-I HC level.

**Figure 3:**
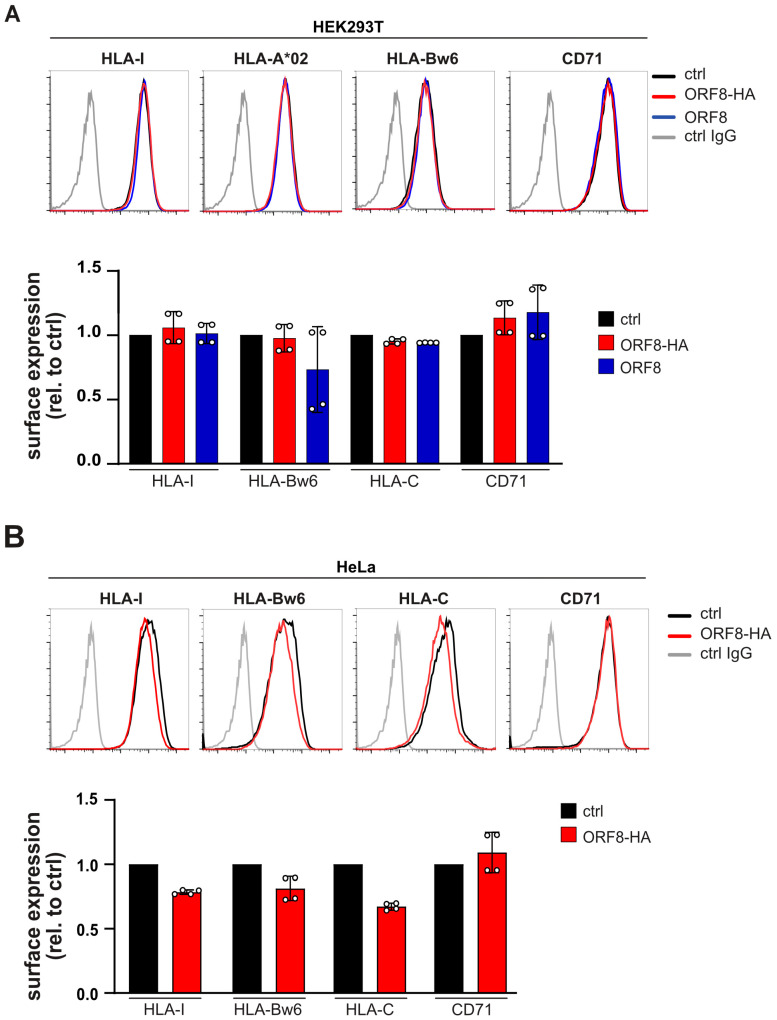
Cells stably expressing ORF8 do not regulate HLA-I. (**A**,**B**) Lentivirally transduced HEK293T (**A**) and HeLa (**B**) cells expressing a control protein, ORF8, or ORF8-HA were analyzed by flow cytometry to determine the cell surface expression level of HLA-I as indicated (upper panels). Fold change of surface expression (MFI) on cells expressing ORF8 or ORF8-HA is shown compared to control cells (lower panels). Dots represent individual values, and bars represent mean values ± SD from four independent experiments. Statistical analysis using a one-way ANOVA did not find any significant changes.

Since potential HLA-I allele-specific effects of ORF8 could be masked in Western blot analysis, we used allele-specific antibodies and assessed surface expression by flow cytometry. This showed only a marginal regulation by ORF8 in HeLa cells and no effect in HEK293T cells (Figure 3A,B). Furthermore, in metabolic co-immunoprecipitation experiments using these cells, we did not detect an interaction between ORF8-HA and HLA-I. By contrast, HA-US11 bound to HLA-I and induced a strong HLA-I downregulation, whereas in ORF8-HA-expressing cells, the level of newly synthesized HLA-I remained equal to those in control cells (Supplementary Material Figure S2B). Notably, during puromycin selection of ORF8-transduced cells, we observed impaired cell growth for up to two weeks, suggestive of an adaptive process in the surviving cells, possibly reducing the ORF8 expression (Supplementary Material Figure S2B). This adaptation may have counteracted potential ORF8-mediated effects on HLA-I expression, explaining the limited changes observed by flow cytometry and metabolic labeling.

### 3.4. High-Level ORF8 Overexpression Leads to Downregulation of HLA-I

**Figure 4:**
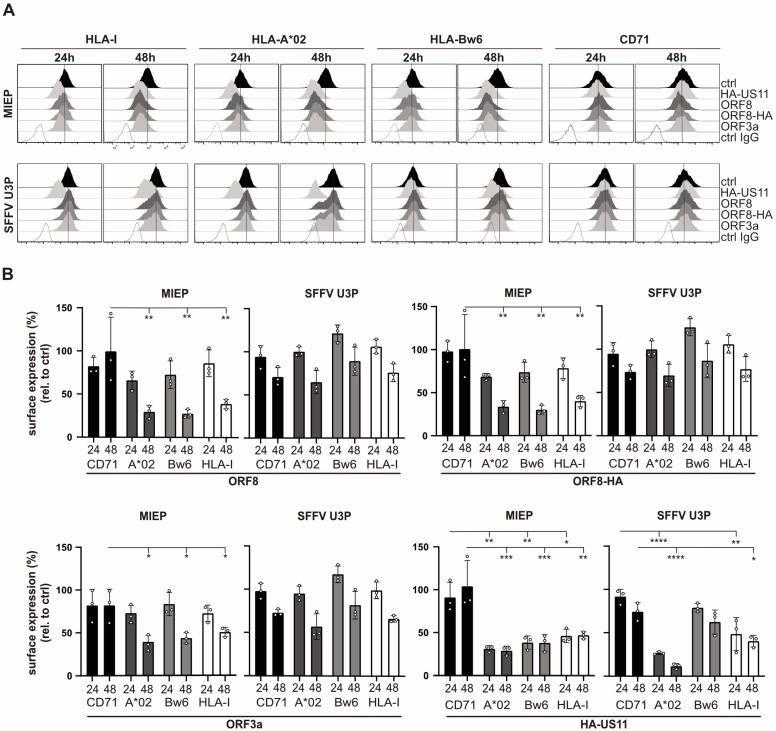
High-level ORF8 overexpression leads to downregulation of HLA-I. (**A**) HEK293T cells were transiently transfected with expression plasmids with the gene of interest controlled by the major CMV IE promoter (MIEP in pIRES-EGFP, upper panel) or the spleen-forming focus virus U3 promoter (U3P in pucIP, lower panel). HLA-I, HLA-A*02:01, HLA-Bw6, and CD71 surface expression was analyzed on EGFP-positive cells at indicated time-points post-transfection. (**B**) Fold change of HLA-I (allotypes as indicated) surface expression (MFI) by ORF8, ORF8-HA, ORF3a, and HA-US11 in A and B was compared to control-transfected cells. Dots represent individual values, and bars represent mean values ± SD from three independent experiments. To determine the specific effect of HLA-I molecules, statistical significance compared to the CD71 expression was calculated using one-way ANOVA test. Only comparisons with *p* < 0.05 are marked in the figure at *p* < 0.05 (*), *p* < 0.01 (**), *p* < 0.001 (***), and *p* < 0.0001 (****).

To circumvent effects from cell adaptation to stress or other cellular processes that ORF8 might target, [37,38,39], we assessed the impact of transient ORF8 expression. ORF8, ORF8-HA, ORF3a, HA-US11, and a control protein were transiently expressed in HEK293T cells using a vector comprising an internal ribosome entry site (*IRES*) followed by *EGFP* to identify transfected cells. While at 24 h post-transfection, no significant effects on HLA-I expression were observed; by 48 h post-transfection, both ORF8 and ORF8-HA caused a substantial reduction (ca 60–70%) in total HLA-I, HLA-A*02:01, and HLA-Bw6 surface expression compared to control-transfected cells (Figure 4A, upper panel). ORF3a was included as an additional SARS-CoV-2 protein since an effect of ORF3a has been observed on HLA-I trafficking [14]. While ORF3a also reduced HLA-I expression, its effect was weaker than that for ORF8. The positive control, HA-US11, strongly inhibited surface expression of all investigated HLA-I already at 24 h post-transfection, indicating that ORF8-mediated regulation occurs with delayed kinetics compared to HA-US11.

We previously reported that US11 preferentially targets HLA-A over HLA-B in HCMV-infected cells [25]. However, in this experimental system, only a small difference in downregulation of HLA-A*02:01 and HLA-Bw6 was observed. Since US11 is known to induce an unfolded protein response (UPR) [40], we speculated that the observed effects may be at least partly influenced by the strong expression driven by the CMV major immediate early promoter (MIEP) in the pIRES–EGFP vector. To assess whether strong overexpression could cause aberrant effects, potentially due to nonspecific protein interactions or activation of UPR, we employed a vector in which the gene of interest is driven by the spleen focus-forming virus U3 promoter (U3P). This vector yielded lower protein levels as compared to the pIRES–EGFP construct (Supplementary Material Figure S3A). Indeed, the choice of vector had a marked impact on HLA-I surface expression (Figure 4A lower panel and Figure 4B). At 48 h post-transfection, on the surface of cells transfected with ORF8 or ORF8-HA encoding plasmids, HLA-A*02:01 downregulation ranged from approximately 30% (MIEP) to 65% (U3P) compared to control-treated cells. For HLA-Bw6, the surface downregulation ranged from 70% (MIEP) to only 10% (U3P) compared to mock-treated cells, demonstrating that a strong expression of both ORF8 and ORF3a is required for a potent HLA-I downregulation (illustrated also in Supplementary Material Figure S3B). US11 also showed interesting promoter-dependent effects. When expressed from the MIEP promoter, HLA-A*02:01 levels were 71% downregulated compared to control, while expression from SFFV U3P resulted in a more pronounced downregulation, reducing 89% from the cell surface. In contrast, HLA-Bw6 expression was reduced from 60% with MIEP to 38% with U3P, consistent with the previously reported US11 specificity in HCMV-infected cells [25]. These findings suggest that strong overexpression of proteins that can trigger UPR [38,40] may induce unspecific regulation of HLA-I. This is consistent with prior data showing that ER stress can lead to downregulation of HLA-I surface expression [41].

### 3.5. Nsp1 Effectively Inhibits Biosynthesis of HLA-I

**Figure 5:**
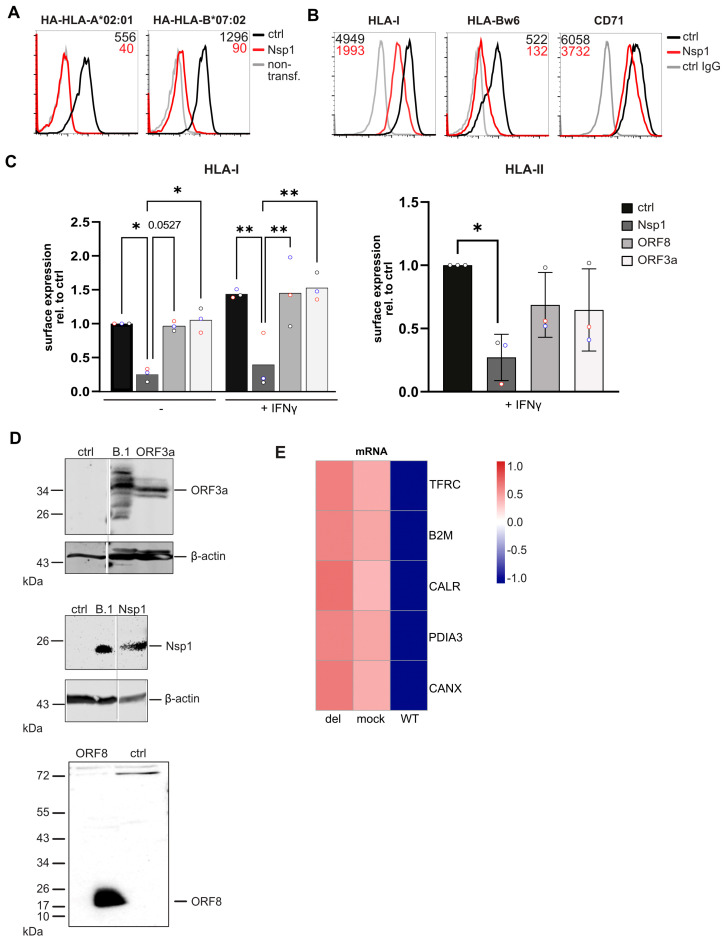
Nsp1 inhibits biosynthesis of HLA-I. (**A**) HEK293T cells were transiently co-transfected with expression plasmids (pucIP) encoding EGFP and either Nsp1 or a control with expression plasmids (pucIP) without EGFP expression encoding HA-tagged HLA-A*02:01 or HLA-B*07:02. At 24 h post-transfection, surface expression of HLA-I molecules was determined on EGFP-positive cells by flow cytometry using an anti-HA antibody. (**B**) HEK293T cells were transiently transfected with expression plasmids (pucIP) encoding EGFP and either Nsp1 or a control. At 24 h post-transfection, expression of indicated surface proteins was determined on EGFP-positive cells by flow cytometry. (**C**) Relative surface expression of HLA-I (left panel) and HLA-II (right panel) was determined on MRC5 fibroblasts transduced with lentiviruses encoding EGFP and, in addition, Nsp1, ORF8, ORF3a, or a control. IFNy was applied where indicated at 12 h post-transduction. At 72 h post-transduction, cell surface expression of indicated proteins was determined by flow cytometry on EGFP-positive cells. Representative histograms are shown in Supplementary Material Figure S4B. Fold change of MFI of transduced cells compared to control cells is shown. Dots represent individual values, and bars represent mean values ± SD from three independent experiments. Statistical significance compared to cells transduced with control vector was assessed using a one-way ANOVA (N = 3). Only comparisons with *p* < 0.05 are marked in the figure at *p* < 0.05 (*), *p* < 0.01 (**). (**D**) Cell lysates of SARS-CoV-2 (B.1)-infected Calu-3 cells and transiently transfected HEK293T cells with an Nsp1, ORF3a, and ORF8 in lentiviral vectors (pucGFP) were analyzed by Western blotting with antibodies as indicated. (**E**) Heatmaps showing the relative mRNA levels at 7 h post-infection of selected genes in cells infected with either wild-type virus (WT) or Nsp1-deletion mutant (del).

The lack of differences in HLA-I regulation by B.1 and B.1.1.7 suggested that, independently of ORF8, another stronger regulatory mechanism exists that is responsible for HLA-I expression during SARS-CoV-2 infection. The non-structural protein Nsp1 has previously been shown to be a potent type I interferon inhibitor and responsible for the viral shutoff of host protein translation [17,19]. To test whether Nsp1 can control biosynthesis of HLA-I, we transiently co-expressed N-terminally HA-tagged HA-HLA-A*02:01 and -B*07:02 with Nsp1 or a control protein (all under control of the SFFV U3 promoter) in HEK293T cells and analyzed surface expression by flow cytometry. Nsp1 completely blocked both HLA-A*02:01 and HLA-B*07:02 expression at the cell surface (Figure 5A; ORF8 did not affect HLA-I in this setup, Supplementary Material Figure S4A). This demonstrated that Nsp1 is highly efficient in abrogating biosynthesis of HLA-I. To determine the effect of Nsp1 on endogenous HLA-I expression, Nsp1 or a control protein was transiently expressed in HEK293T cells. Also in this setting, Nsp1 efficiently suppressed HLA-I expression and, to a somewhat lesser extent, CD71 expression (Figure 5B).

To analyze HLA-II, we transduced MRC-5 fibroblasts with lentiviruses, driving Nsp1, ORF8, ORF3a, or a control protein expression in front of an *IRES* and *EGFP* sequence with the SFFV U3 promoter. Cells were stimulated with IFNy 12 h post-transduction. At 72 h post-transduction, surface expression of HLA-I and HLA-II was determined by flow cytometry on EGFP-positive cells. In Nsp1-expressing cells, HLA-I was strongly downregulated, both with and without IFNy stimulation, by 75% and 64%, respectively (Figure 5C). In contrast to this, no HLA-I downregulation was observed in ORF8- or ORF3a-expressing cells. IFNy-induced expression of HLA-II was efficiently inhibited by Nsp1, reducing expression by 77%, as compared to control cells. Surprisingly, in cells expressing ORF8 and ORF3a, HLA-II surface expression was robustly downregulated by 54% in presence of ORF3a and 31% in presence of ORF8. The roles of ORF8 and ORF3a in regulating HLA-II expression remain to be elucidated. Expression of proteins from the lentiviral vectors was verified by Western blot analysis (Figure 5D).

To further investigate whether Nsp1 can modulate the expression of genes required for HLA-I biosynthesis in SARS-CoV-2-infected cells, we analyzed a bulk RNA-seq dataset from SARS-CoV-2-infected cells at 7 h post-infection, comparing wild-type virus with a virus lacking Nsp1 [17]. Although transcripts for several classical HLA-I antigen processing genes (*HLA-A/B/C*, *TAP1*, *TAP2*, and *TAPBP*) were not detected in sufficient amounts in this dataset, transcripts for multiple ER-resident HLA-I co-factors were identified. Expression of β_2_m (*B2M*), calreticulin (*CALR*), ERp57 (*PDIA3*), and calnexin (*CANX*) was markedly downregulated in wild-type-infected cells, but restored to near-normal levels in the absence of Nsp1 (Figure 5E). This effect was observed also for CD71 (*TFRC*). These data support a model in which Nsp1 broadly suppresses host gene expression early during infection, including key components required for HLA-I maturation and surface expression.

## 4. Discussion

To escape T-cell immunity, viruses have evolved diverse immune evasion mechanisms [42]. In this study, we investigated SARS-CoV-2-encoded strategies and identified Nsp1 as a potent inhibitor of HLA-I and HLA-II biosynthesis, potentially limiting T-cell-mediated control.

In SARS-CoV-2 infection experiments, plasma membrane HLA-I was strongly downregulated on N-positive cells, whereas non-infected bystander cells showed elevated HLA-I levels compared to mock-treated controls, consistent with previous reports [43]. Upregulation of HLA-I in bystander cells is likely driven by interferon signaling induced by infected cells [44]. Re-analysis of an available single-cell RNA sequencing dataset from SARS-CoV-2-infected human lung organoids [32] showed transcriptional induction of genes involved in HLA antigen presentation in both bystander and infected cells. However, this induction was attenuated in infected cells compared to bystander cells, indicating that while some antiviral signaling persists, it is impaired in the presence of active viral replication.

In addition, we found that SARS-CoV-2 infection not only blocked the basal and virus-induced HLA-I expression, but also markedly inhibited IFNγ-induced upregulation of both HLA-I and HLA-II surface expression, indicating a broad suppression of IFNγ-driven HLA expression in infected cells. Although SARS-CoV-2 is well documented to antagonize type I interferon responses [12,17,45], our data support a model in which the virus also impairs type II interferon-induced antigen presentation, most likely through a post-transcriptional mechanism.

ORF8 was the first SARS-CoV-2 factor reported to impair MHC-I surface expression [13]. Due to ongoing positive selection, some successful SARS-CoV-2 variants have emerged that lack ORF8 expression [46]. One such variant, the Alpha strain B.1.1.7, was included in our analysis but showed no difference in HLA-I suppression compared to the ORF8-expressing strain, suggesting that ORF8 is not essential for this phenotype. The continued suppression of HLA-I, despite the absence of ORF8 expression in the Alpha variant, has also been reported by others [43,47]. While transient high-level ORF8 expression reduced HLA-I surface levels, moderate or stable expression had minimal effects. ORF8 has been linked to ER stress responses [39], a condition known to suppress MHC-I expression [41,48]. Interestingly, ORF8 was found to induce ER stress by binding calnexin and BiP (HSPA5), key ER chaperones involved in quality control, but notably, the same study found no evidence of direct MHC-I regulation [39]. These findings are consistent with the possibility that high-level ORF8 overexpression induces elevated ER stress, which may secondarily affect MHC-I expression, rather than reflecting a specific immune evasion strategy. Early reports linking ORF8 to MHC-I degradation [13,47] resulted in the exclusion of the protein from vaccine studies. Based on our data, we conclude that ORF8 is unlikely to play a major role in inhibiting HLA-I antigen presentation in vivo, though it may still serve as a relevant antigenic target [49].

In contrast to ORF8, ectopic expression of Nsp1 using various conditions and cells markedly reduced HLA-I surface levels and completely blocked IFNγ-induced upregulation. This highlights a potent inhibition of newly synthesized HLA-I across alleles and cell types, impacting both basal and interferon-induced expression. This is not surprising since Nsp1 blocks global host gene expression by disrupting translation and inducing degradation of host transcripts, thereby also efficiently blocking IFN-induced gene expression [17,18,19]. Importantly, because newly synthesized HLA-I molecules are suppressed, the presentation of newly translated viral proteins, including defective ribosomal products (DRiPs), is also likely impaired. Since DRiPs constitute an important source of peptides for antigen presentation [50], Nsp1-mediated suppression of HLA-I may represent a highly effective strategy to evade CD8+ T-cell recognition.

Nsp1 has been extensively studied, and a SARS-CoV-2 mutant lacking Nsp1 was previously analyzed for effects on host transcript levels and stability [17]. Bulk RNA-seq data from that study revealed that key ER-resident components of the HLA-I presentation pathway, including *B2M*, *CALR*, *CANX*, and *PDIA3*, were significantly downregulated in cells infected with wild-type virus. In contrast, expression of these genes was restored in cells infected with the Nsp1-deletion mutant, directly implicating Nsp1 in the suppression of HLA-I antigen presentation. Strong downregulation of *B2M* and of *TAP1* and *PSMB9* mRNA has been described also by others [10]. Although these findings support a key role for Nsp1 in impairing antigen presentation, a limitation of our study is the lack of a direct comparison between wild-type and Nsp1-deletion virus under our specific experimental conditions. As a result, we cannot precisely estimate the extent to which Nsp1 contributes to the observed phenotype in our system.

Taken together, analysis of RNA-seq data supports our observations and a model in which Nsp1 blocks the HLA-I presentation pathway. Translational shutoff of host genes is an established immune evasion strategy among DNA viruses, such as alphaherpesviruses, which encode the highly conserved UL41 protein to inhibit host protein synthesis. This was found to reduce MHC-I and MHC-II expression and CD8+ T-cell recognition of infected cells [51,52,53]. We now show that an RNA virus can deploy a similar strategy. Moreover, Nsp1-mediated translational shutoff is conserved across the *Betacoronavirus* family [22], suggesting that Nsp1-mediated evasion of T-cell responses may be a widespread feature of the *Betacoronavirus* family.

## Figures and Tables

**Table 1:** Primer sequences used for cloning.

Primer	Sequence
ORF8_for ORF8_rev	5′-GCATGCTAGCATGAAATTTCTTGTTTTCTTAGG-3′ 5-GCATGGATCCTTAGATGAAATCTAAAACAACACG-3′
**ORF8-HA_for** **ORF8-HA_rev**	5′-GCATGCTAGCATGAAATTTCTTGTTTTCTTAGG-3′ 5′-GCATGGATCCTTATGCGTAATCTGGAACATCGTATGGGTAGATGAAATCTAAAACAACACG-3′
ORF3a_for ORF3a_rev	5′-GCATGCTAGC ATGGATTT GTTTATGAGA ATCTTCACAA-3′ 5′-GCATGGATCCTTACAAAGGCACGCTAGTAG-3′
nsp1_for nsp1_rev	5′-GCATGCTAGC ATGGAGAGCCTTGTCCCT-3′ 5′-GCATGGATCCTTACCCTCCGTTAAGCTCACG-3′

## Data Availability

The original data presented in the study are openly available (See Material and Methods section) or available from the corresponding author on request.

## Supplementary Materials

The following supporting information can be downloaded at: https://www.mdpi.com/article/10.3390/v17081083/s1, Figure S1: Supplement Figure S1 (related to Figure 2); Figure S2: Supplement Figure S2 (related to Figure 3); Figure S3: Supplement Figure S3 (related to Figure 4); Figure S4: Supplement Figure S4 (related to Figure 5).
