# Supplementary material for: SARS-CoV-2 Nsp1 Is a Major Suppressor of HLA Class I and Class II Expression
Source: Viruses. 2025 Aug 5;17(8):1083. doi: 10.3390/v17081083 (PMC12390542; doi:10.3390/v17081083)
Supplement: Supplementary file 1 [file viruses-17-01083-s001.zip › viruses-3754867-supplementary.pdf]

Supplemental information

SARS-CoV-2 Nsp1 is a major suppressor of HLA class I and class II expression

Ivo Schirmeister[1,2,3], Nicolas Eckert[1,2], Sebastian Weigang[1,2], Jonas Fuchs[1,2], Lisa Kern[1,2,3], Georg Kochs[1,2], Anne Halenius[1,2]

[1]Institute of Virology, Medical Center University of Freiburg, Freiburg, Germany; [2]Faculty of Medicine, University of Freiburg, Freiburg, Germany; [3]Faculty of Biology, University of Freiburg, Freiburg, Germany

Methods

Immunoprecipitation and autoradiography

Cells stably expressing ORF8-HA or HA-US11 were cultured in 6-well plates and metabolically labeled with EasyTag Express [^{35}S]-methionine/cysteine protein labeling mix (Perkin Elmer) at a concentration of 0.2 mCi/mL for 2 hours. After labeling, cells were washed and lysed in 1% (w/v) digitonin lysis buffer (Calbiochem) containing 140 mM NaCl, 20 mM Tris (pH 7.6), and cOmplete protease inhibitor cocktail (Roche). Immunoprecipitation was performed at 4° C using an overhead tumbler, followed by incubation with protein A Sepharose beads (GE Healthcare). Beads were washed with a stepwise NaCl gradient to increase stringency. Bound complexes were eluted by heating in sample buffer containing 150 mM DTT at 95° C for 5 minutes. Immunoprecipitated proteins were resolved by gradient SDS-PAGE. Gels were fixed, dried, and analyzed by autoradiography using a Typhoon FLA 7000 scanner following phosphor screen exposure.

Supplemental Figures

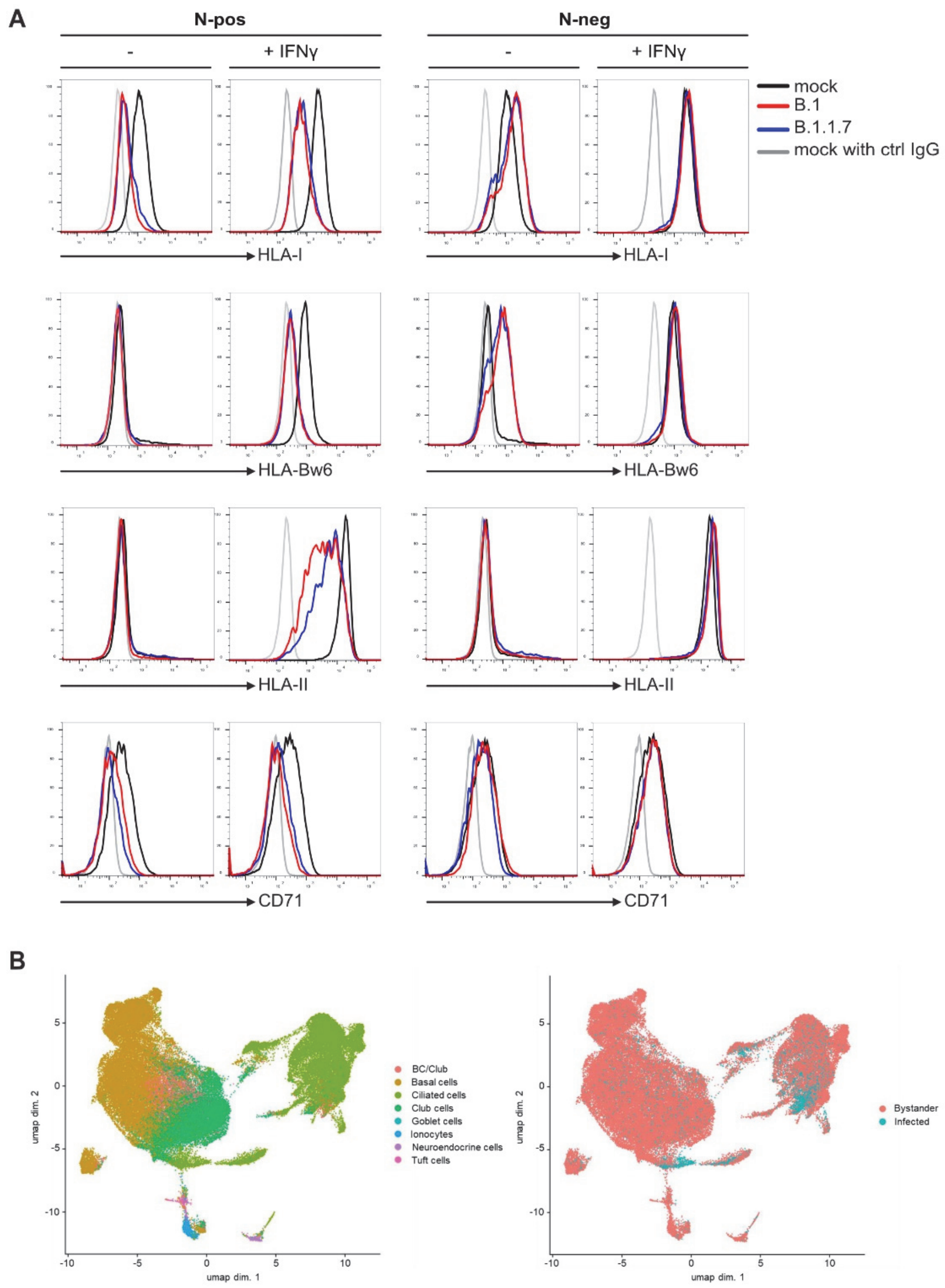

Figure S1 (related to Fig. 2). (**A**) Calu-3 cells were infected with SARS-CoV-2 or SARS-CoV-2-ΔORF8 with an MOI of 0.5. Simultaneously, IFNγ was applied to the cells. At 30 h p.i. cells were analyzed by flow cytometry with antibodies as indicated. Cell populations were gated according to an intracellular N-staining. (**B**) Cluster analysis via Seurat and Harmony algorithm applied to single-cell transcriptomes of human bronchial epithelial cells (HBECs). Each dot corresponds to one individual cell. Cells were highlighted with respect to distinct HBEC cell types (left) and status of infection (right).

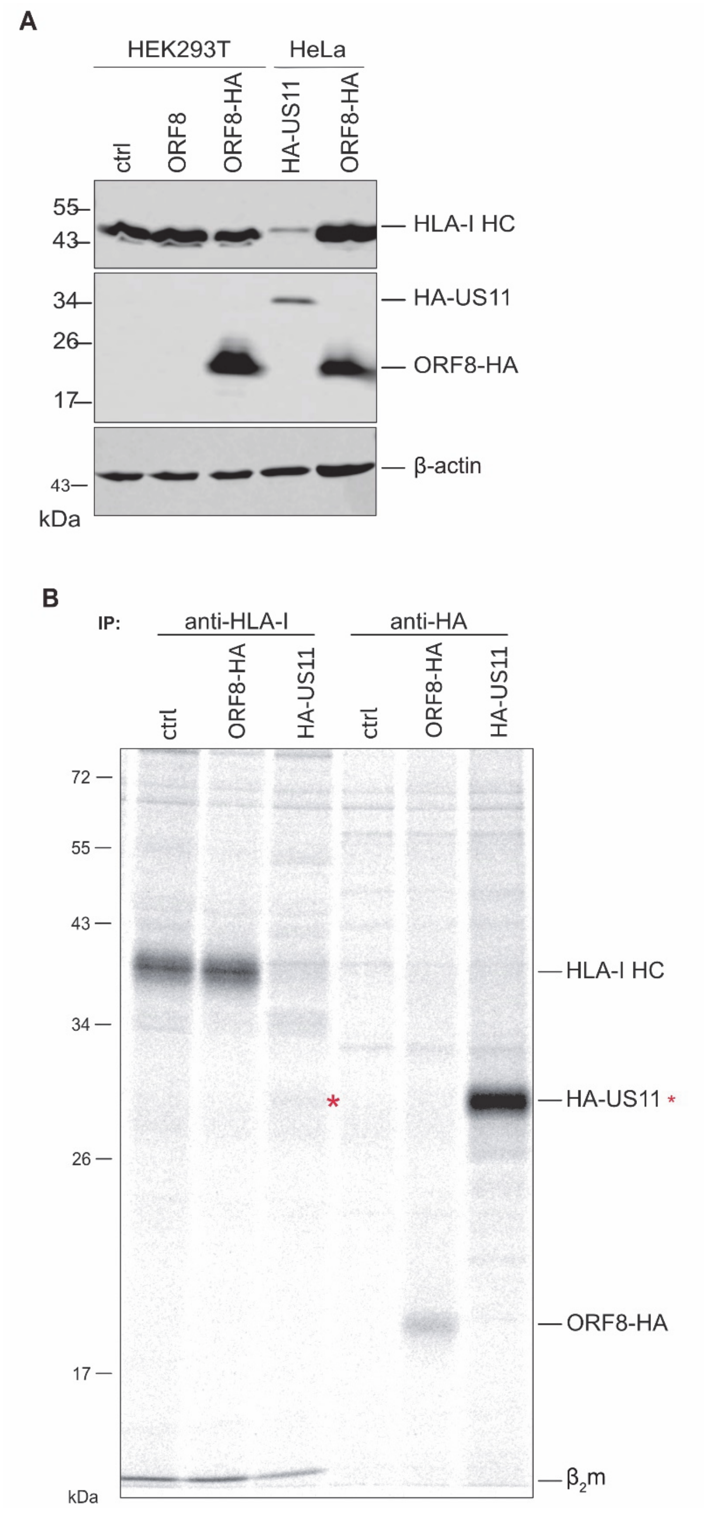

Figure S2 (related to Fig. 3). (**A**) HEK293T cells were lentivirally transduced as indicated. Cell lysates were prepared and analyzed by Western blot with the mAb HC10 (HLA-I HC), anti-HA, and anti-β-actin. (**B**) HeLa cells stably expressing ORF8-HA or US11-HA were metabolically labeled (^{35}S-methionine/cysteine) for 2 h. Cell lysates were prepared and immunoprecipitation with anti-HLA-I (W6/32) and anti-HA antibodies were performed. Proteins were separated by SDS-PAGE and detected via autoradiography.

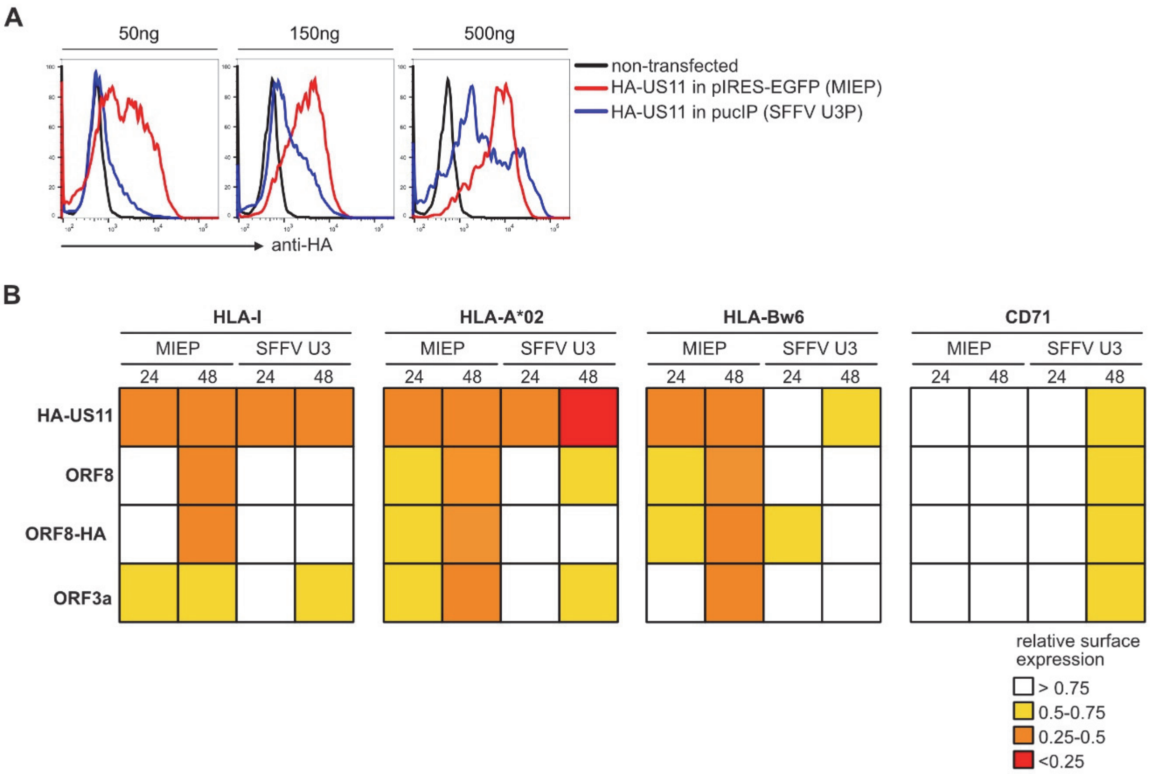

Figure S3 (related to Fig. 4). (**A**) HEK293T cells were transiently transfected with either pIRES-EGFP (MIEP promoter) or pucIP (SFFV U3 promoter) expression vectors with various DNA amounts as indicated. At 24 h post-transfection HA-US11 expression level was determined by an intracellular anti-HA stain. (**B**) Using the data from Fig. 4B, a color-coded scheme of the relative surface expression of HLA-I and CD71 proteins is illustrated.

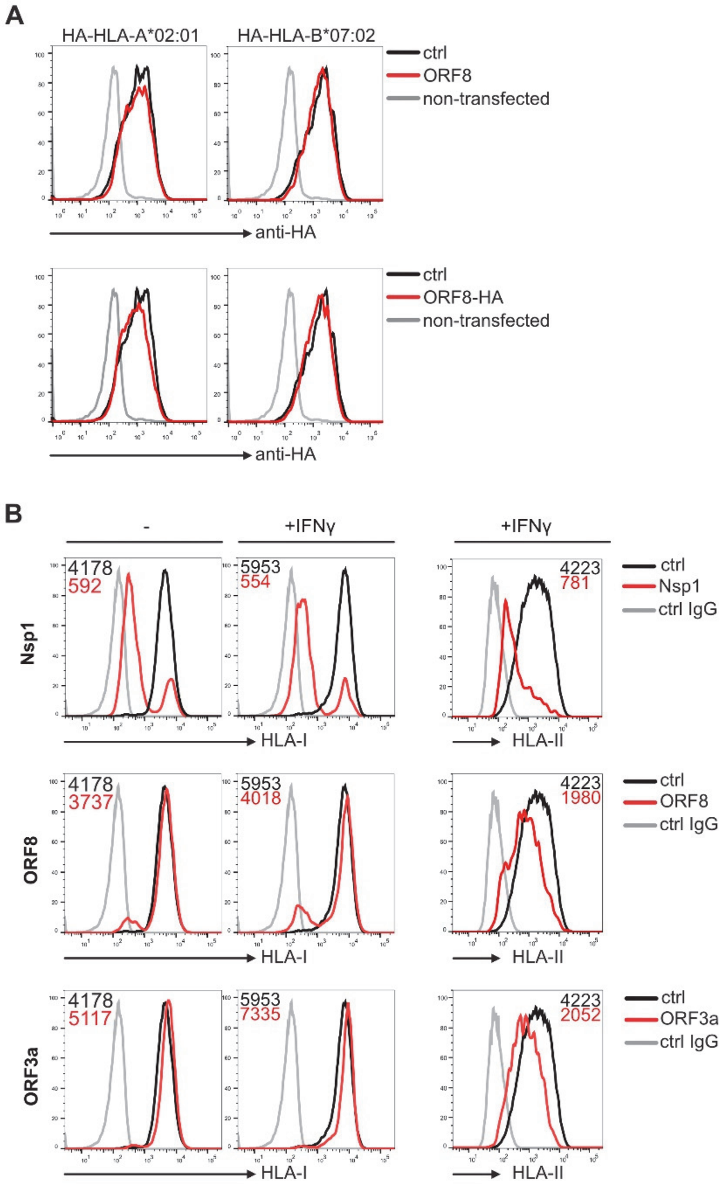

Figure S4 (related to Fig. 5). (**A**) ORF8 and ORF8-HA (in pucGFP) were transiently co-transfected with HA-HLA-A*02:01 or HA-HLA-B*07:02 into HEK293T cells. Surface expression of newly synthesized HLA-I (anti-HA) was determined 24 h post-transfection via flow cytometry. (**B**) surface expression of HLA-I (left panel) and HLA-II (right panel) was determined on MRC5 fibroblasts transduced with lentiviruses encoding EGFP, and, in addition, Nsp1, ORF8, ORF3a, or a control. IFNy was applied where indicated at 12 h post-transduction. At 72 h post-transduction cell surface expression of indicated proteins was determined by flow cytometry on EGFP positive cells.
